# Ck2-Dependent Phosphorylation Is Required to Maintain Pax7 Protein Levels in Proliferating Muscle Progenitors

**DOI:** 10.1371/journal.pone.0154919

**Published:** 2016-05-04

**Authors:** Natalia González, James J. Moresco, Felipe Cabezas, Eduardo de la Vega, Francisco Bustos, John R. Yates, Hugo C. Olguín

**Affiliations:** 1 Departamento de Biología Celular y Molecular, Facultad de Ciencias Biológicas, Pontificia Universidad Católica de Chile, Santiago 8331150, Chile; 2 Department of Chemical Physiology, The Scripps Research Institute, La Jolla, CA 92037, United States of America; University of Rome La Sapienza, ITALY

## Abstract

Skeletal muscle regeneration and long term maintenance is directly link to the balance between self-renewal and differentiation of resident adult stem cells known as satellite cells. In turn, satellite cell fate is influenced by a functional interaction between the transcription factor Pax7 and members of the MyoD family of muscle regulatory factors. Thus, changes in the Pax7-to-MyoD protein ratio may act as a molecular rheostat fine-tuning acquisition of lineage identity while preventing precocious terminal differentiation. Pax7 is expressed in quiescent and proliferating satellite cells, while its levels decrease sharply in differentiating progenitors Pax7 is maintained in cells (re)acquiring quiescence. While the mechanisms regulating Pax7 levels based on differentiation status are not well understood, we have recently described that Pax7 levels are directly regulated by the ubiquitin-ligase Nedd4, thus promoting proteasome-dependent Pax7 degradation in differentiating satellite cells. Here we show that Pax7 levels are maintained in proliferating muscle progenitors by a mechanism involving casein kinase 2-dependent Pax7 phosphorylation at S201. Point mutations preventing S201 phosphorylation or casein kinase 2 inhibition result in decreased Pax7 protein in proliferating muscle progenitors. Accordingly, this correlates directly with increased Pax7 ubiquitination. Finally, Pax7 down regulation induced by casein kinase 2 inhibition results in precocious myogenic induction, indicating early commitment to terminal differentiation. These observations highlight the critical role of post translational regulation of Pax7 as a molecular switch controlling muscle progenitor fate.

## Introduction

Satellite cells (SCs) are tissue specific stem cells present in the adult skeletal muscle and largely responsible for its regenerative capacity [1–3]. In resting or uninjured muscle, SCs reside between the plasma membrane and the basal lamina of the myofiber, in a quiescent state [4,5]. Upon local or systemic stimuli, SCs become activated, proliferate, migrate and induce the expression of the muscle regulatory transcription factor MyoD [6]. Following a transient phase of robust cell division, these lineage committed progenitors (adult myoblasts) induce the expression of another member of the MyoD family, myogenin, triggering the process of terminal differentiation, leading to new myofiber formation and/or myofiber repair [7]. Critical for the maintenance of their stem cell nature, SCs must balance self-renewal with the potential to give rise to lineage committed progenitors [8]. Whereas it is well stablished that muscle differentiation is under the transcriptional control of the MyoD family, the molecular regulation of SC maintenance and renewal has recently begun to unveil. In this regard, the transcription factor Pax7 is required for SC specification [9–11] and function [12,13]. Evidence from our group and others indicate that Pax7 play dual roles in SCs [14]. While Pax7 can initiate the myogenic program by inducing *myoD* and/or *myf-5* transcription [15–17], it can also repress MyoD activity and the induction of terminal differentiation [18–20]. In this context, Pax7-to-MyoD protein ratio appears to regulate SC fate (i.e. self-renewal vs. differentiation). Accordingly, Pax7 is rapidly down regulated by the ubiquitin ligase Nedd4 and the proteasome system, in differentiating but not in proliferating muscle progenitors [21]. Nedd4 sub-cellular localization is differentially regulated in muscle progenitors. Importantly, accumulation of Nedd4 in the nucleus (via inhibition of exportin 1-dependent nuclear export) is sufficient to down regulate Pax7, inducing myogenin expression. Conversely, Nedd4 knockdown prevents terminal muscle differentiation [21]. It is unclear however, how Nedd4 localization is controlled nor the mechanisms coordinating Pax7 retention in self-renewing progenitors and down regulation in differentiating cells.

Here we provide evidence that Pax7 is phosphorylated by casein kinase 2 (CK2) in proliferating myoblasts. Site-directed mutagenesis and CK2 loss of function suggest that Pax7 phosphorylation prevents its down regulation via the ubiquitin-proteasome system.

## Materials and Methods

Procedures involving animal tissues were performed according to National Commission for Science and Technology (CONICYT) guidelines and approved by Facultad de Ciencias Biológicas of Pontificia Universidad Católica de Chile bioethics and biosecurity committee.

### Cell culture

C2C12 (ATCC^®^ CRL-1772^™^) and C3H10T1/2 (ATCC^®^ CCL-226^™^) cell lines were cultured in Dulbecco’s modified Eagle’s medium (DMEM) (Life technologies) supplemented with 10% fetal bovine serum (FBS) (Hyclone) and 1% penicillin/streptomycin (Life technologies) at 37°C with 5% CO2. C3H10T1/2 subjected to myogenic conversion, were differentiated using DMEM 2% FBS. Adult primary myoblasts were obtained as described [21] and maintained in growth medium F12-C (Life technologies) with 15% horse serum (HS) (Hyclone), 1% penicillin/streptomycin and 1 nM FGF-2 at 37°C with 5% CO2. When required, cells were incubated with 10–25 μM MG132 (Cell Signaling), 1μM epoxomicin (Sigma-Aldrich), 75–125 μM TBB (Abcam), 10–50 μM TBCA (Calbiochem) or dimethyl sulfoxide (DMSO) (Sigma-Aldrich) for the indicated times.

### Pax7 point mutants

pCDNA3-myc-NLSPax7WT plasmid [20] was used to produce Pax7 single point mutants using Quick Change multi-site directed mutagenesis XL kit (Agilent Technologies). To disrupt phosphorylation, serine was changed to alanine using the following primer pairs: 5’-cgtctggatgagggcgcagatgtggaatcag-3’ and 5’-ctgattccacatctgcgccctcatccagacg-3’ to Pax7 S201A (Pax7 AS) mutant; 5’-ggctcagatgtggaagcagaacccgacctc-3’ and 5’-gaggtcgggttctgcttccacatctgagcc-3’ to Pax7 S205A (Pax7 SA) mutant; 5’-ggcgcagatgtggaagcagaacccgacctc-3' and 5’-gaggtcgggttctgcttccacatctgcgcc-3' to Pax7 S201A/S205A (Pax7 AA) mutant. To mimic phosphorylation, serine was changed to aspartic acid using the following primer pairs: 5'-cgggttctgattccacatcatcgccctcatccagacggttc-3' and 5'-gaaccgtctggatgagggcgatgatgtggaatcagaacccg-3' to Pax7 S201D (Pax7 DS) mutant; 5'-gggggaggtcgggttcatcttccacatctgagccc-3' and 5'-gggctcagatgtggaagatgaacccgacctccccc-3' to Pax7 S205D (Pax7 SD) mutant; 5'-cagggggaggtcgggttcatcttccacatcatcgccctcatccagacggtt-3' and 5'-aaccgtctggatgagggcgatgatgtggaagatgaacccgacctccccctg-3' to Pax7 S201D/S205D (Pax7 DD) mutant.

For localization assays, pCDNA3-myc-Pax7WT, pCDNA3-myc-Pax7AS, pCDNA3-myc-Pax7SA and pCDNA3-myc-Pax7AA plasmids were generated by subcloning from pCDNA3-myc-NLS to pCDNA3-myc [21], using BamHI/XhoI restriction sites. For expression assays, C3H10T1/2 cells in 12 well plates were transfected using Lipofectamine 3000 (Life technologies) with 1μg pRSV-MyoD [19], 1 μg pCDNA3-myc-NLSPax7WT or point mutants and 100ng EYFP-MEM (Clontech) as a transfection control. Cells were transfected with half Pax7 cDNA amounts to achieve comparable protein expression of WT and mutants in others experiments.

### RNA isolation, reverse transcription and qPCR

Total RNA was extracted using a RNASolv isolation system following the manufacturer's instructions. First, the genomic DNA was degraded using DNAse I at 25°C for 15 min. The RNAs were then quantified using Tecan equipment (with the NanoDrop module). The first strand of cDNA was synthesized using 0.5 μg of RNA in 20 μl of reaction buffer by reverse transcription using RevertAid^™^ H Minus M Mulv 200 U/μl and random primers at 42°C for 60 min. PCR was performed to verify reverse transcription using Taq polymerase and a 0.4 μl aliquot of cDNA with specific primer pairs. The sequences of primers used for qPCR were as follows: mouse Pax7 5’-CACCCCTTTCAAAGACCAAA-3’ (Forward) and 5’TGCTTGAAGTTCCTGCTCCT-3’ (reverse); mouse 18S 5’- GAGCGAAAGCATTTGCCAAG-3’ (Forward) and 5’- GGCATCGTTTATGGTCGGAA-3’ (Reverse). The real-time PCR reactions were conducted using SYBR Green master mix according to the manufacturer's instructions. A 7500 real-time PCR system (Applied Biosystems) was used to measure the relative RNA levels and the data were analysed using 7500 system SDS software. qPCR analysis was performed using a relative quantification mathematical model, as previously described [22].

### Western blotting

Whole cell extracts were obtained by disruption in modified RIPA lysis buffer (50 mM Tris-HCl pH 7.4, 150 mM NaCl, 1% IGEPAL, protease and phosphatase inhibitors (Merck) and incubated at 4°C for 20 min, followed by centrifugation at 15200 rpm for 10 min. Proteins (20–30 μg) were separated into 10% SDS-PAGE gels and transferred to polyvinylidene difluoride (PVDF) membranes (Thermo Fisher Scientific). Membranes were blocked with 5% (wt/vol) nonfat powdered milk in TBS-T (20 mM Tris, pH 7.4; 100 mM NaCl; 1% Tween-20) and incubated with the following primary antibodies and dilutions: mouse monoclonal anti-Pax7 (Developmental Studies Hybridoma Bank) at 1:10; mouse monoclonal anti-myc-tag (9B11) (Cell Signaling) at 1:1000; rabbit monoclonal anti-GFP (E385) (Abcam) at 1:10000, mouse monoclonal anti-GAPDH (EMD Millipore) at 1:10000; rabbit MultiMab^™^ anti-phospho-CK2 Substrate (pS/pT)DXE (Cell Signaling) at 1:1000. Anti-mouse IgG and anti-rabbit IgG HRP-conjugated secondary antibodies (Cell Signaling) were used at 1:5000, and HRP activity was visualized using SuperSignal West Pico Chemiluminescent Substrate or SuperSignal West Dura Extended Duration Substrate (Thermo Fisher Scientific). Relative densitometry was measured using ImageJ software.

### Immunofluorescence

Cells were fixed in 4% paraformaldehyde in PBS, permeabilized with 0.5% Triton X-100 and blocked with 3% BSA in PBS. Primary antibodies and dilutions were used as follows: mouse monoclonal anti-Pax7 (Developmental Studies Hybridoma Bank) at 1:5; rat monoclonal anti-MyoD (5F11) (Millipore) at 1:100; mouse monoclonal anti-myogenin (F5D) (Developmental Studies Hybridoma Bank) at 1:5; chicken anti-Syndecan-4 [23] at 1:500; rabbit monoclonal anti-active caspase-3 (C92-605) (BD Pharmingen) at 1:100. The following secondary antibodies were used at 1:500: goat anti-mouse Alexa 594, goat anti-rabbit Alexa 594, goat anti-mouse Alexa 488, donkey anti-rat 488 (Life technologies) and donkey anti-chicken-AMCA (Jackson). Cell nuclei were stained with Hoechst 33342 (Life technologies) and Vectashield (Vector Laboratories) was used as mounting media. Images were acquired using an IX71 microscope (Olympus) equipped with a QICam FAST QImaging camera and MoticBA410 microscope with a Moticam Pro 252B camera. Images were processed using Illustrator (Adobe).

### In vitro phosphorylation assay

Purified GST-Pax7WT, GST-Pax7AS, GST-Pax7SA and GST-Pax7AA (5 μg) were incubated with or without 100 ng of CK2 (New England BIolabs) in kinase buffer provided with the enzyme (50 mM Tris-HCl, 10 mM MgCl2, 0.1 mM EDTA, 2 mM DTT, 0.01% Brij 35) supplemented with 100 μM ATP (New England BIolabs) for 30 min at 30°C. Reactions were loaded into SDS-PAGE gel and analyzed by Western blotting.

### Cell-Based Ubiquitination

Ubiquitination assay was performed as previously described [21]. Briefly, C2C12 cells were transfected using Transit X2 (Mirus) with pCDNA3-Pax7d and pCMV7-myc-6xHis-Ub. 24 hours later, cells were incubated with 100 μM TBB or DMSO for 12h, and 12.5 μM MG132 was added for the last 6 hours before harvesting. Cells were resuspended in buffer A2 (6 M guanidine chloride, 0.1 M Na2HPO4/NaH2PO4, 10 mM immidazole, pH 8.0) incubated with 50 μl of Ni-NTA agarose (QIAGEN) for 3 hours at room temperature. After washing, proteins were eluted with buffer ETI (25 mM Tris-HCl, 300 mM imidazole, pH 6.8) for 10 minutes at room temperature and analyzed by SDS-PAGE and Western blotting.

### Ubiquitin-Mediated Fluorescence Complementation

Plasmids for bi-molecular fluorescence complementation (BiFC) were obtained as described previously [21]. C2C12 cells were transfected using Transit X2 with plasmids encoding C-terminus fragment of Venus Fluorescent protein (VC155) fused to Pax7WT, Pax7AA, Pax7DS, Pax7DD or bFos and N-terminus fragment of Venus (VN155-I152L) fused to ubiquitin or bJun as specified. Gap43-mRFP was included as a transfection marker. 24 hours post transfection, cells were incubated with 25 μM MG132 and 125 μM TBB or DMSO as indicated, for 6 hours before fixation. Fluorescence complementation was evaluated in the epifluorescence microscopy mentioned.

### Myogenic conversion and reporter assay

C3H10T1/2 cells were induced to myogenic conversion by co-transfecting pRSV-MyoD vector in all experiments. For repressing myogenic conversion, cells were co-transfected with pCDNA3-myc-NLSPax7WT, Pax7AS, Pax7SA or Pax7AA and pRSV-MyoD in 2:1 ratio as previously described [19]. Differentiation was induced 24 h after transfection for 96 h, and then cells were fixed and subjected to immunofluorescence.

To evaluate Pax7-point mutants transcriptional activity, C3H10T1/2 cells were transfected with the Pax3/7 specific reporter gene *6xPRS9-Luc* [19] and pCDNA3-myc-NLSPax7WT, Pax7AS, Pax7SA or Pax7AA as indicated. CMV-LacZ vector for constitutive ß-galactosidase expression was co-transfected in order to normalize luciferase activity. Pax7FKHR was used as positive control for reporter activation, as described previously [19]. 24 hours post transfection, whole cell lysates were collected and luciferase and β-galactosidase activities were determined using the Dual-Light System (Applied Biosystems) following manufacturer’s instructions. Luminescence was measured in Tecan Infinite M200 PRO microplate reader.

### Mass spectrometry

C2C12 cells were transfected with pCDNA3-myc-NLSPax7WT using Lipofectamine 2000 (Life technologies) and were maintained in growth medium or differentiated for 48 hours before harvesting. Nuclear extracts were obtained as described [21] and subjected to immunoprecipitation with anti-myc-tag (9B11) sepharose bead conjugate (Cell Signaling) at 1:50. Proteins were eluted 3 times with 0.1 M glicine-HCl, pH 2.5 for 5 min, and neutralized with 0.5 M Tris-HCl, pH 7.4, 1.5 M NaCl. Proteins were reduced with 5 mM Tris(2-carboxyethyl)phosphine hydrochloride (Sigma-Aldrich, St. Louis, MO, product C4706) and alkylated with 55 mM 2-Chloroacetamide (Fluka Analytical, product 22790). Proteins were digested for 18 h at 37°C in 2 M urea 100 mM Tris pH 8.5, 1 mM CaCl_2_ with 2 μg trypsin (Promega, Madison, WI, product V5111). MudPIT aalysis was performed using an Agilent 1100 quaternary pump and a Thermo Velos LTQ-Orbitrap using an in-house built electrospray stage [24].

Protein and peptide identification and protein quantitation were done with Integrated Proteomics Pipeline—IP2 (Integrated Proteomics Applications, Inc., San Diego, CA. http://www.integratedproteomics.com/). Tandem mass spectra were extracted from raw files using RawExtract 1.9.9 [25] and were searched against a Uniprot mouse protein database) with reversed sequences using ProLuCID [26,27]. The search space included all fully-tryptic and half-tryptic peptide candidates with fixed modification of 57.02146 on C and variable modification of 79.9663 on STY. Peptide candidates were filtered using DTASelect [28], with these parameters -p 2 -y 1—trypstat—fp .01—modstat—extra—pI -DM 10—DB—dm -in -m 1.

### Statistics

All quantitative data are presented as mean ± SEM, unless indicated. Number of independent experiments are specified in figure legends. Statistical analysis was performed with GraphPad Prism software, using one-way ANOVA followed by Tukey’s post hoc test to determine significance. A p ≤ 0.05 was considered significant.

## Results

### Pax7 is phosphorylated by CK2 in myoblasts

As shown recently, post translational regulation of Pax7 levels via the ubiquitin-proteasome system is critical to allow myogenic progression to terminal differentiation [21]. It remains unknown, however, how Pax7 levels are maintained in self-renewing muscle progenitors. Based on our previous studies we hypothesized that a different post translational modification could stabilize Pax7 protein in myoblasts.

**Fig 1:**
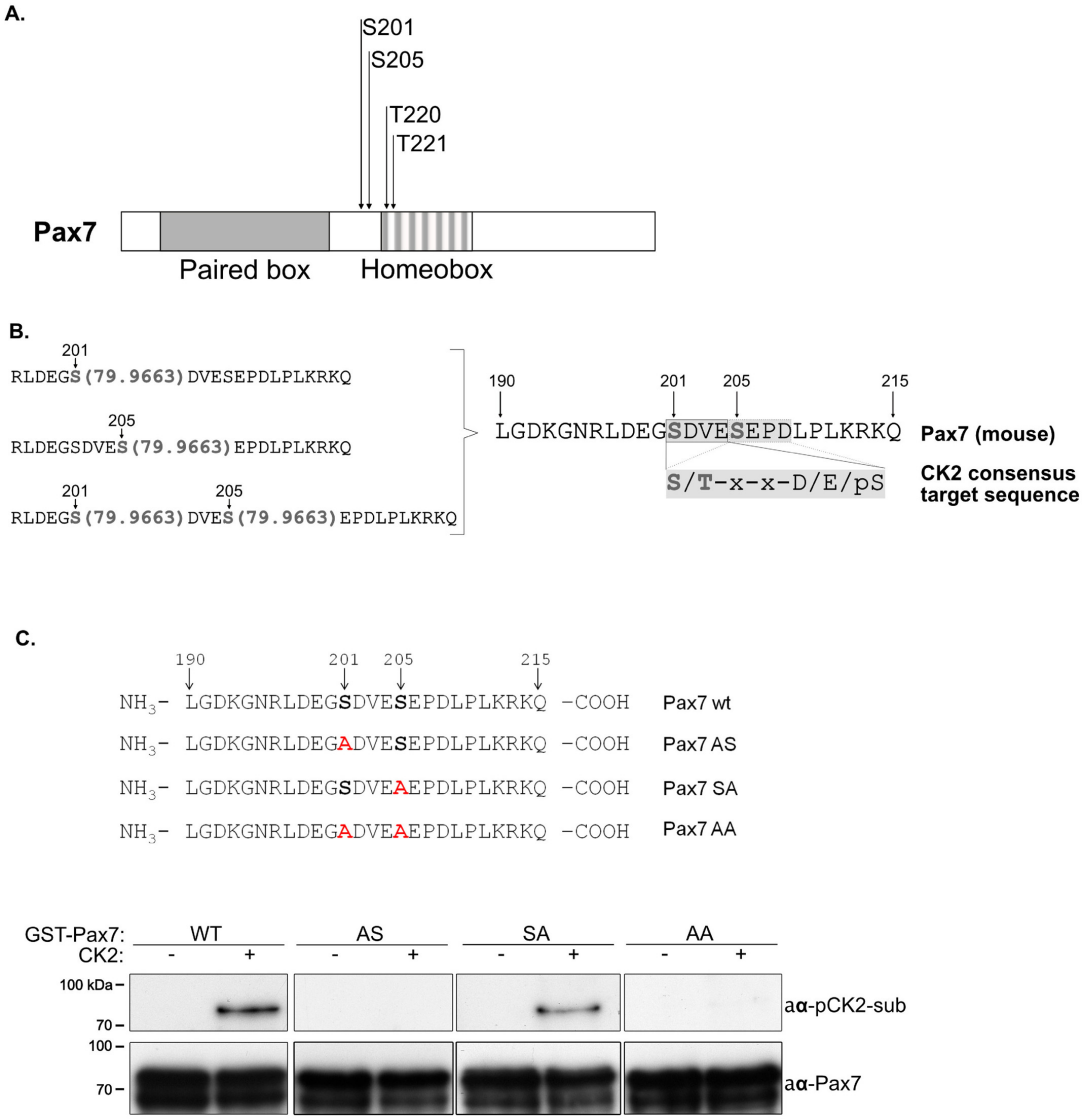
Pax7 is phosphorylated by casein kinase II (CK2) in proliferating myoblasts. (A) Schematic representation of *in silico* analysis of mouse Pax7 sequence, depicting Pax7 domains and aminoacids with the highest probabilities to be phosphorylated by serine/threonine kinases. (B) Mass spectrometry analysis of myc-Pax7 expressed in proliferating C2C12 myoblasts, shows three Pax7 phospho-peptides, indicated by a 79.9663 increase in mass. Pax7 serine 201 and serine 205 are located in two consecutive consensus sequences for CK2 phosphorylation (*S/T-x-x-D/E/pS). (C) Upper panel shows schematic representation of Pax7 point mutations (see Materials and Methods) at the indicated sites. Lower panel shows *in vitro* phosphorylation assays using purified WT GST-Pax7 protein (WT) or GST-Pax7 mutants (AS, SA, AA, respectively), in presence or absence of purified CK2. Only WT and SA are detected with anti-phospho-CK2 substrate antibody (anti-pCK2-sub) by Western Blot, indicating that serine 201 is directly phosphorylated by CK2.

*In silico* inspection of Pax7 primary sequence revealed four potential phosphorylation sites, ranked with the highest probabilities to be modified. Interestingly, these candidate sites were located in close proximity to each other and were predicted to be substrates for serine/threonine kinases (Fig 1A). In order to determine if any of the candidate sites were modified *in vivo*, we expressed low levels of myc-tagged Pax7 in C2C12 myoblasts. Cells were maintained either in proliferating or differentiating culture conditions for 48 h, prior to myc-Pax7 immunoprecipitation from whole-cell lysates. Samples were digested, enriched for phosphopeptides using TiO2 affinity chromatography and analyzed by mass spectrometry. As shown in Fig 1B, phosphoproteomic analysis revealed the presence of two phosphorylated serine residues (S201 and S205). Both of these sites were predicted *in silico* (Fig 1A). Importantly, Pax7 phosphorylation at S201 and S205 was detected only in samples obtained from proliferating myoblasts; we could not identify phosphorylated sites from differentiating samples. Further inspection of the identified phosphopeptides revealed that Pax7 S201 and S205 were located in two consecutive casein kinase 2 (CK2) consensus sites (Fig 1B). In order to determine if these sites were directly modified by CK2, site-directed mutagenesis was performed (serine to alanine substitution; Fig 1C) in order to prevent phosphorylation of Pax7 at S201 (S201A; Pax7-AS), S205 (S205A; Pax7-SA) or both (S201A/S205A; Pax7-AA). Wild type and phospho-mutant GST-Pax7 proteins were subjected to *in vitro* phosphorylation assays in the presence or absence of recombinant CK2 enzyme. Modified products were detected by Western Blot using an anti-phospho CK2-substrates antibody (anti-pCK2subs). As shown in Fig 1C, only Pax7 and Pax7-SA were identified by the anti-pCK2subs antibody. Since no products were detected in the absence of CK2 or Pax7, these results indicate that Pax7 is phosphorylated by CK2 at S201. A recently published study described Pax7 phosphorylation at S201 [29], although the molecular consequences differed substantially (see Discussion).

### Phosphorylation by CK2 regulates Pax7 protein stability in proliferating cells

**Fig 2:**
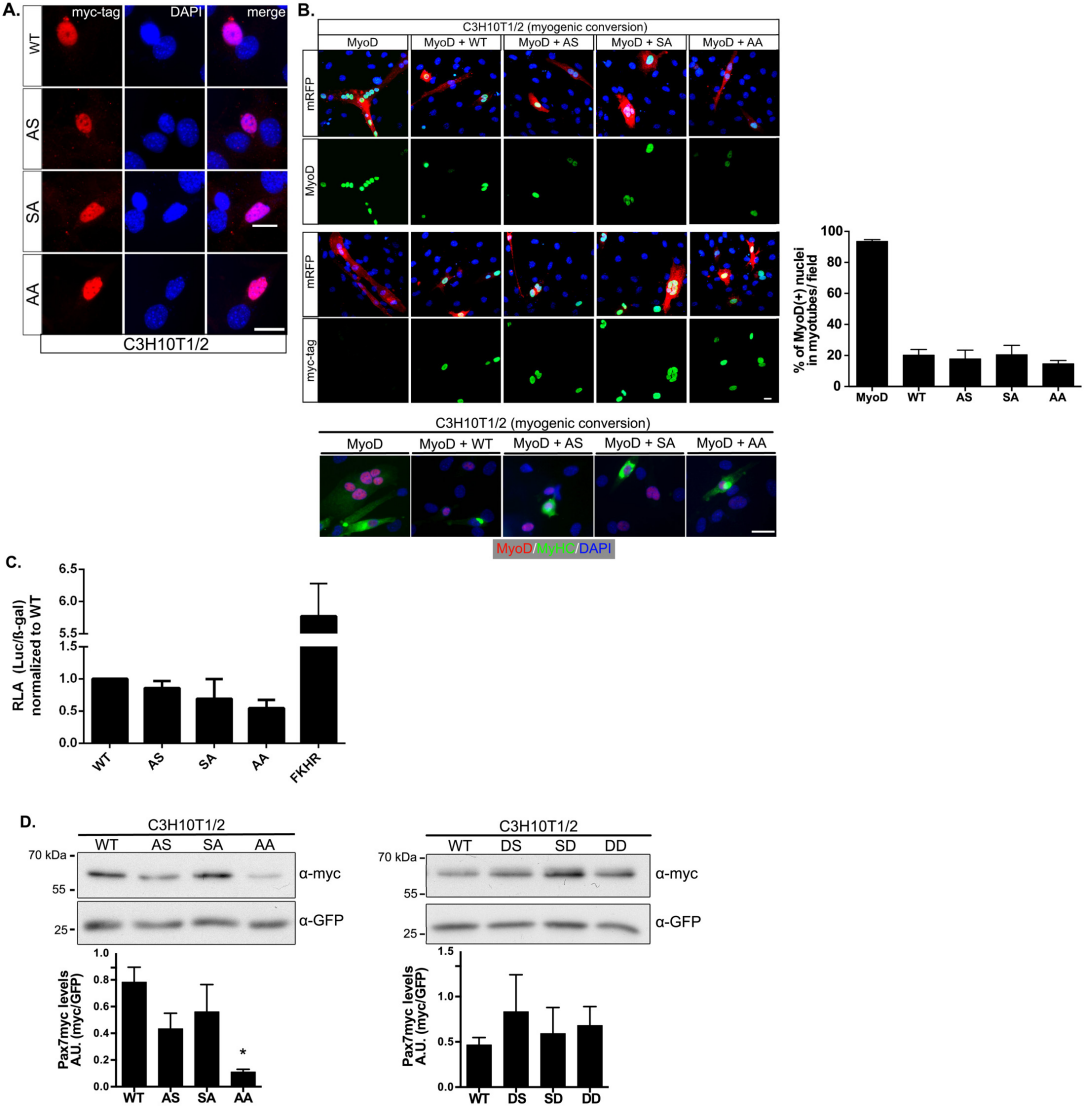
Disruption of Pax7 phosphorylation results in lower Pax7 protein levels. (A) Pax7 localization is not affected by S201/S205 phosphorylation. Immunofluorescence analysis of C3H10T1/2 cells transfected with myc-Pax7-WT, myc-Pax7-AS, myc-Pax7-SA or myc-Pax7-AA shows nuclear localization (DAPI) of all Pax7 variants (myc). Scale bar = 10μm. (B) the ability of each Pax7 mutant to repress MyoD-dependent myogenic conversion of C3H10T1/2 cells, was evaluated by immunofluorescence. All Pax7 mutants repressed myotube formation resembling the effect of Pax7-WT. Panel depicts MyoD and myc-Pax7 expression in transfected mRFP(+) cells. Quantification of three representative experiments, evaluating myotube formation by fusion index (i.e. myotube associated nuclei/ total MyoD+ or myc+ nuclei; considering ≥3 nuclei in mRFP+ cells as a myotube) is shown. Lower panel: the percentage of MyHC associated MyoD(+) nuclei was also inhibited upon Pax7 and Pax7 phospho-mutants co-expression. Bar = 10μm. (C) Transcriptional activity of Pax7-WT or Pax7 phospho-mutants, was evaluated using the *6xPRS9-luc* reporter gene in C3H10T1/2 cells [20]. CMV-LacZ expression vector was co-transfected and β-galactosidase activity was used to normalize luciferase activity. The graph represents the relative luciferase activity (RLA) (luciferase/β-galactosidase) / Pax7-WT activity ratio; mean±SEM, n = 3. No significant differences between Pax7-WT and mutants activity was observed. (D) Phosphorylation status of S201/S205 affects Pax7 protein levels. Upper panels: Western Blot analysis of myc-tagged Pax7 point mutants expression in C3H10T1/2 cells. GFP was used as transfection/loading control. Lower panels show quantification of myc-Pax7/GFP ratio normalized to Pax7-WT; mean±SEM, n = 3 (left), n = 4 (right); ANOVA, * p<0.05. Pax7-AA protein levels are significantly lower than Pax7-WT. Pax7-DS and Pax7-DD phospho-mimetic mutants exhibit higher expression levels compared to Pax7-WT.

S201 and S205 are located between two functional Pax7 domains, the octapetide and the homeobox-DNA binding domain (HD). Based on studies evaluating deletion of specific Pax7 domains [19], we tested Pax7 phospho-mutants for expression, subcellular localization, and anti-myogenic activity. Nuclear localization was not altered upon expression in C3H10T1/2 cells (Fig 2A). Interestingly, although deletion of the HD domain eliminates the ability of Pax7 to repress muscle differentiation, Pax7 phospho-mutants repressed the MyoD-dependent myogenic conversion in C3H10T1/2 cells to levels comparable to WT Pax7. Specifically, Fig 2B depicts the decreased formation of multinucleated cells (mRFP (+) cells) upon co-expression of Pax7 and Pax7 phospho-mutants. Similar results were observed when analyzing the expression of muscle specific proteins such as myosin heavy chain (Fig 2B, lower panel). In this context, we have described that Pax7 transcriptional activity is dispensable to repress MyoD [19]; accordingly, reporter-gene assays revealed no difference between WT Pax7 and the phospho-mutant proteins (Fig 2C). The most noticeable difference was observed when comparing protein levels upon expression in myogenic and non-myogenic cell lines. Pax7-AS and AA mutants showed consistently lower protein levels when compared to WT or Pax7-SA (Fig 2D). Accordingly, phospho-mimetic Pax7 mutants (DS, SD and DD; see Materials and Methods) and WT Pax7 expression levels were not significantly different (Fig 2D). Moreover, Pax7-DS and DD mutants exhibited a tendency towards higher protein expression, although statistical significance was not achieved due to the variability inherent to transient transfections.

**Fig 3:**
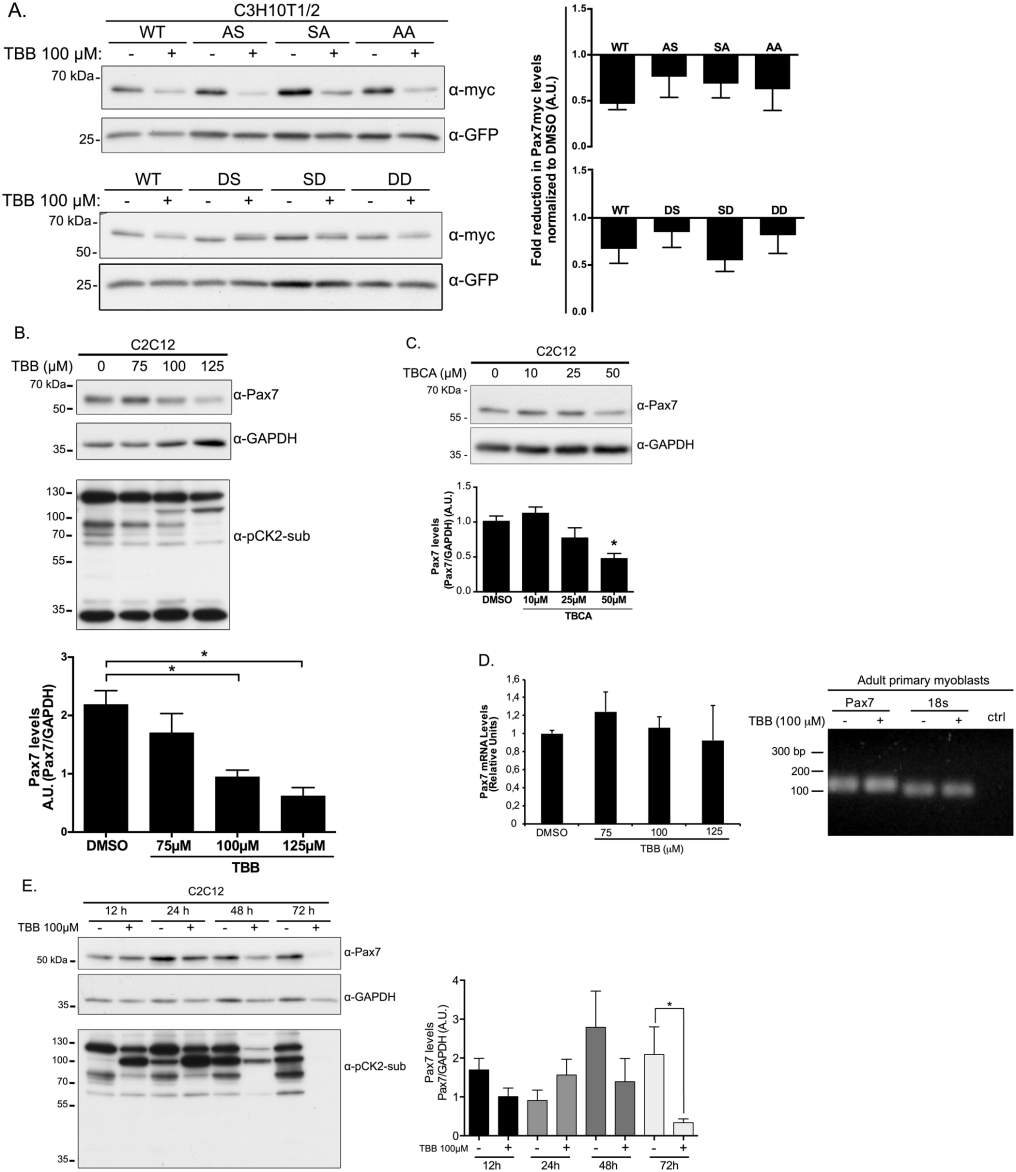
CK2 regulates Pax7 stability in proliferating myoblasts. (A) C3H10T1/2 cells transfected with myc-Pax7-WT or mutants were treated with DMSO or 100 μM of CK2 inhibitor (TBB) for 6 hours prior to lysis and Western Blot analysis. GFP was used as transfection/loading control. Right panels show quantification of fold reduction in myc-Pax7 levels (myc/GFP) for each treatment compared to vehicle (DMSO); mean±SEM, n = 5 (upper), n = 4 (lower). Pax7-DS and Pax7-DD phospho-mimetics exhibit enhanced stability upon CK2 inhibition compared to other phospho-mutants. Proliferating C2C12 cells were incubated with DMSO, TBB (B) or TBCA (C) at the indicated concentration for 6 hours. Endogenous Pax7 levels were analyzed by Western Blot using GAPDH as loading control. Anti-phospho-CK2 substrate antibody was used as a control of TBB treatment. (B)-(C), Lower panels show quantification of Pax7/GAPDH ratio in relative units; mean±SEM, n = 4; ANOVA, * p<0.05. Pax7 protein levels are significantly reduced with 125 μM TBB for 6 hours in proliferating C2C12 cells. (D) (left panel) qPCR analysis determining relative Pax7 mRNA expression upon CK2 inhibition as performed in (B). mean±SEM, n = 3. (Right panel) RT-PCR analysis of Pax7 mRNA expression upon CK2 inhibition in adult primary myoblasts (n = 3). (E) Proliferating C2C12 cells were incubated with DMSO or TBB for 12, 24, 48 and 72 hours, adding fresh doses every 24 hours. Lower panel shows quantification of fold reduction in Pax7 levels (Pax7/GAPDH) for each treatment compared to vehicle (DMSO); mean±SEM, n = 3.

To test if CK2 activity was directly involved in the regulation of Pax7 protein levels, C3H10T1/2 cells expressing Pax7 constructs were treated with the CK2 inhibitor 4,5,6,7-tetrabromobenzotriazole (TBB) and Pax7 expression was evaluated by Western blotting. Although at different magnitudes, WT Pax7 and phospho-mutant protein levels were significantly decreased upon TBB treatment (Fig 3A, upper panels). Interestingly, Pax7-DS and DD phospho-mimetics showed the lowest protein reduction upon CK2 inhibition, suggesting enhanced stability compared to WT Pax7 (Fig 3A, lower panels). To test the effect of inhibiting CK2 activity on endogenous Pax7 levels, C2C12 myoblasts were cultured in proliferating conditions, in the presence of increasing concentrations of TBB for 6 h. As shown in Fig 3B, endogenous Pax7 protein decreased in a TBB dose-dependent manner. Similar Pax7 reduction was observed using a different CK2 inhibitor, Tetrabromocinnamic acid (TBCA) (Fig 3C). To directly determine if the reduction in Pax7 levels upon CK2 inhibition corresponded to a post-translational effect, Pax7 mRNA expression was evaluated by qPCR (Fig 3D). Pax7 mRNA remained unchanged independently of the TBB doses. Moreover, Pax7 mRNA remained constant upon CK2 inhibition in adult primary myoblasts, as determined by RT-PCR (Fig 3D).

Additionally, increasing the interval of CK2 inhibition at a single TBB concentration, significantly decreased Pax7 levels as a function of incubation time (Fig 3E).

Together, these results suggest that Pax7 is phosphorylated in proliferating myoblasts by CK2 activity, inducing its stabilization.

### CK2 activity prevents Pax7 ubiquitination in proliferating cells

**Fig 4:**
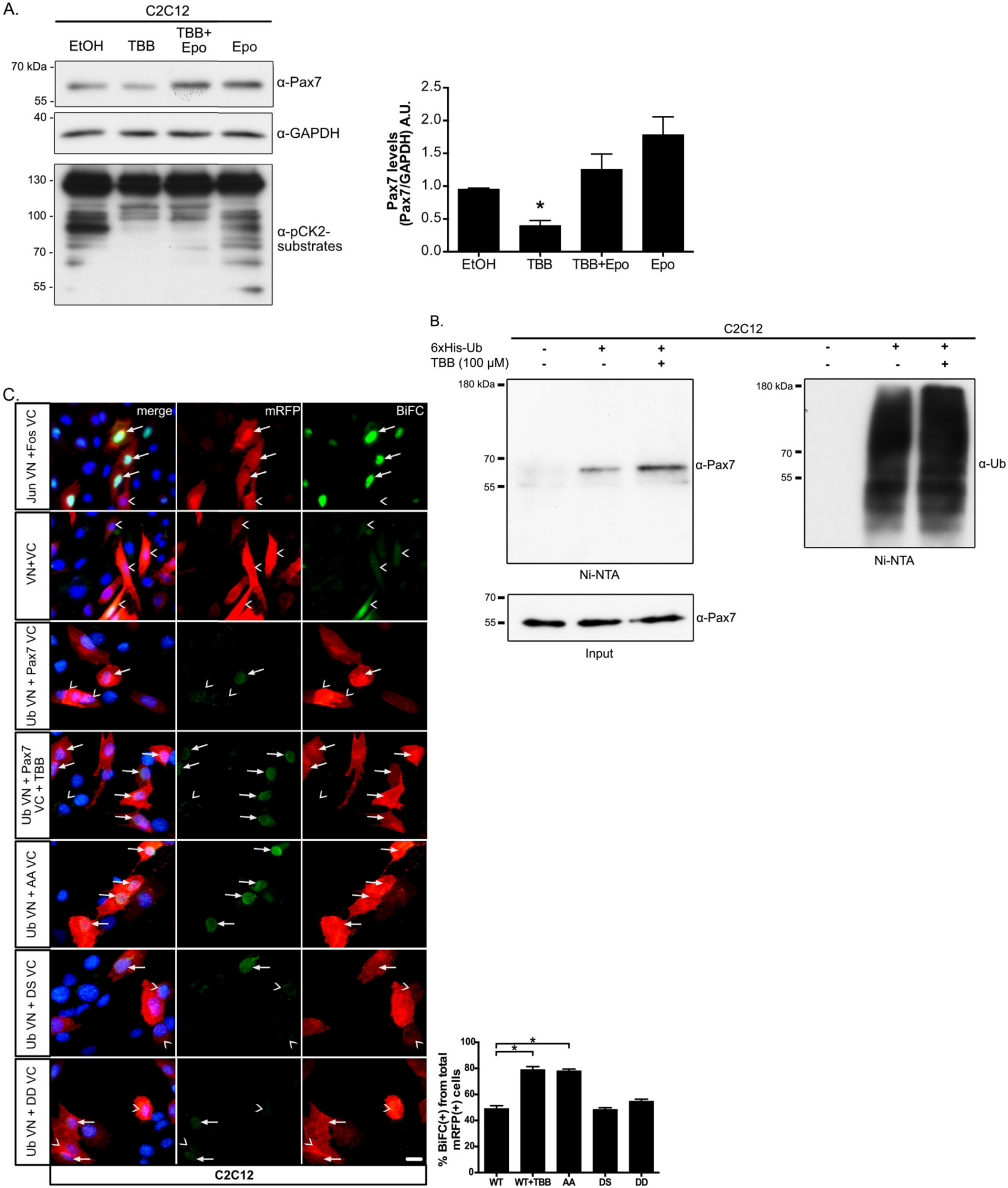
CK2 inhibition in proliferating myoblasts increases Pax7 ubiquitination and its proteasome-dependent degradation. (A) TBB-induced Pax7 decline is prevented by concomitant proteasome inhibition. Proliferating C2C12 cells were treated as indicated with TBB 125 μM and/or 1 μM of the proteasome inhibitor epoxomicin (Epo) for 6 hours and analyzed by Western blotting. GAPDH was used as loading control and anti-phospho-CK2 substrate antibody was used as a control of TBB treatment. Right panel shows quantification of protein levels (Pax7/GAPDH) normalized to control (DMSO); mean±SEM, n = 3; ANOVA, * p<0.05. (B) TBB increases Pax7 ubiquitination in proliferating myoblasts. C2C12 cells were transfected with Pax7 and myc-6xHis-ubiquitin, treated with DMSO or 100 μM TBB for 12 hours and 12.5 μM MG132 was added for the last 6 hours before cell lysis. Denaturing Ni-NTA pull-down, followed by Western Blot shows higher levels of ubiquitinated Pax7 in TBB treated cells. Inputs = 10% of cell extracts prior to Ni-NTA pull-down. (C) Disruption of Pax7 phosphorylation by CK2, increases its ubiquitination *in vivo*. C2C12 myoblasts were transfected with the specified constructs for bimolecular fluorescence complementation (BiFC) and treated with 25 μM MG132 and 125 μM TBB or DMSO as indicated, for 6 hours before fixation. Interaction of bFos-VC and bJun-VN was included as a positive control for BiFC. Transfection of VC and VN was used as a negative control, exhibiting non-specific diffuse fluorescence signal. Arrows indicate positive BiFC and arrowheads shows transfected cells (mRFP positive cells) without complementation. Quantification of BiFC positive cells from total mRFP positive population mean±SEM, n = 3; ANOVA, * p<0.05.

We have recently showed that Pax7 protein levels are negatively regulated in differentiating cells via ubiquitin-ligase Nedd4 and the ubiquitin-proteasome system (UPS) [21]. It remains unclear however, the molecular events maintaining Pax7 levels in proliferating muscle progenitors. As shown by Bustos and cols. (2015), differential Nedd4 sub cellular localization appears as an attractive regulatory switch controlling Pax7 in adult primary myoblasts. In C2C12 myoblasts, Nedd4 appears to be constantly shuttling between the cytoplasm and the nucleus, highlighting the presence of additional mechanisms preventing Nedd4 from targeting Pax7 in proliferating conditions [21]. Given the results presented above, phosphorylation by CK2 represented a plausible candidate to promote Pax7 stability. In this context, we hypothesized that inhibition of CK2 activity would result in enhanced Pax7 ubiquitination. We first tested this idea in C2C12 myoblasts cultured in proliferating conditions in the presence or absence of TBB and the proteasome inhibitor epoxomycin. As expected, decrease in Pax7 levels observed upon CK2 inhibition was prevented by concomitant proteasome inhibition (Fig 4A). Since epoxomycin did not changed the global effect of TBB over CK2 substrates, this result suggested that CK2 activity prevented Pax7 regulation by the UPS. Next, we determined the ubiquitination status of Pax7 in C2C12 myoblasts expressing 6xHis-myc-ubiquitin, cultured in proliferation conditions in the presence or absence of TBB. Affinity purification of ubiquitinated proteins followed by Western blotting, showed that levels of ubiquitinated Pax7 increased upon inhibition of CK2 (Fig 4B). Additionally, we studied the effect of inhibiting Pax7 phosphorylation by bimolecular fluorescence complementation assay (BiFC) as described previously [21]. Supporting the findings described above, basal levels of Pax7 ubiquitination were significantly increased (~1.5 fold) in cells treated with TBB or expressing the Pax7 AA phosphor-mutant (Fig 4C). Accordingly, Pax7 phospho-mimetic mutants (Pax7 DS and Pax7 DD), showed basal ubiquitination levels which further supports the concept that Pax7 is a phospho-protein in proliferating myoblasts.

**Fig 5:**
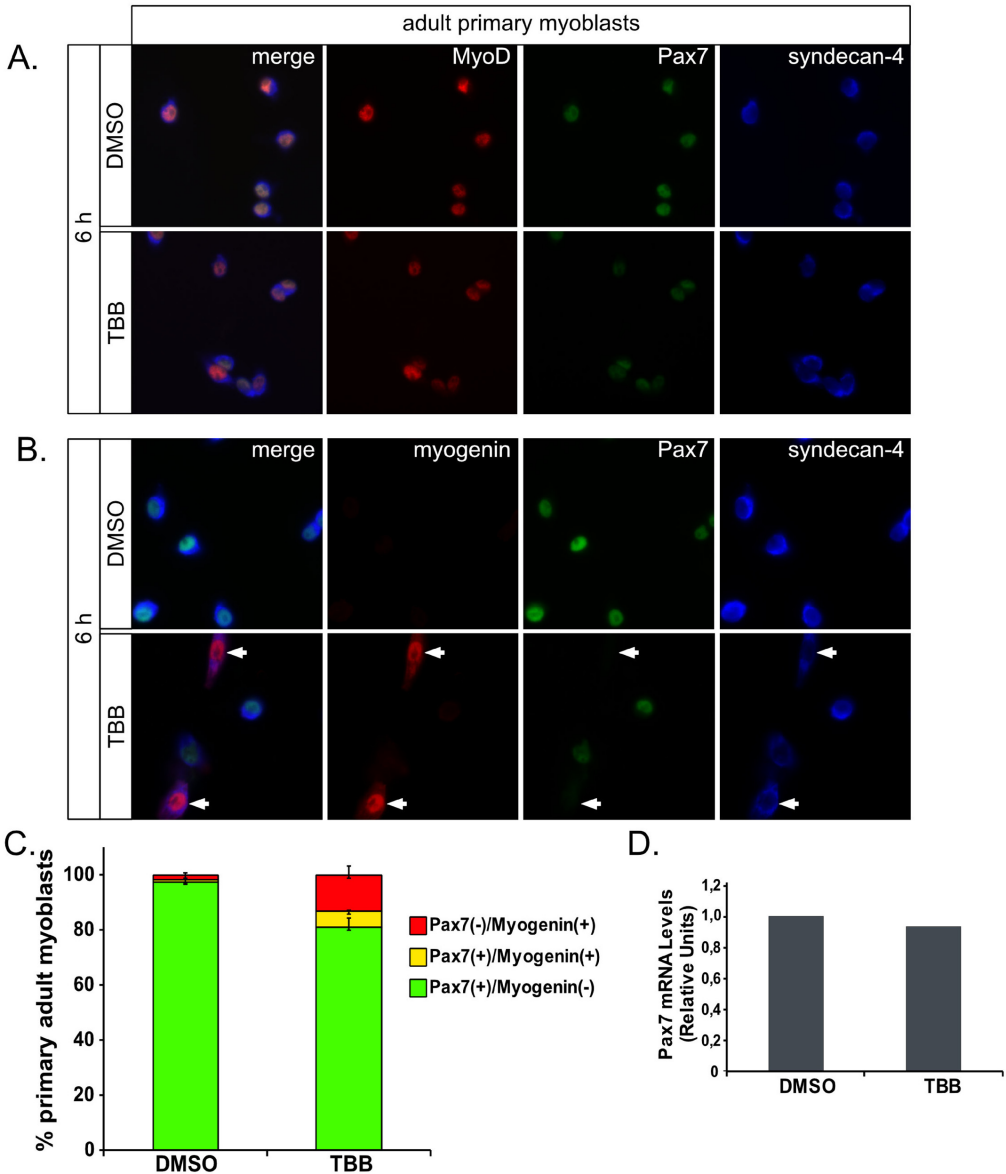
CK2 inhibition in proliferating cells resulted in accelerated expression of differentiation markers. 24 h after isolation, mouse primary myoblasts were incubated with 100 μM TBB or DMSO for 6 hours before fixation. Indirect immunofluorescence for Pax7, Syndecan-4 (A, B), MyoD (A), Myogenin (B) was performed as indicated. TBB treatment results in a significant increase in the percentage of cells expressing reduced Pax7 levels, while expressing high levels of myogenin (arrows). (C) Quantification of cell subpopulations present in (B), shows a ~10-fold increase in the percentage of Pax7(-)/ myogenin (+) cells (i.e. differentiating cells) with a concomitant decrease in the percentage of proliferating Pax7(+)/myogenin(-) sub population. mean±SEM, n = 3. (D) qPCR analysis determining relative Pax7 mRNA expression upon CK2 inhibition (sample pool obtained from one experiment performed as in A and B).

Importantly, CK2 inhibition in adult primary myoblats resulted in decreased Pax7 levels, concomitant to the induction of myogenin in the same cell subpopulation–despite that proliferating culture conditions were maintained- thus suggesting precocious commitment to terminal differentiation (Fig 5A–5C). Although, not statistically significant, pPCR analysis of relative Pax7 mRNA is in line with previous observations, revealing no change in *Pax7* transcription despite the significant effect at the protein level (Fig 5D).

Together, the results presented above indicate that CK2 appears to prevent UPS down regulation of Pax7 protein specifically in proliferating cells, thus preventing precocious commitment to terminal differentiation.

## Discussion

The balance between Pax7 and MyoD proteins appears to act as a molecular rheostat, fine-tuning acquisition of lineage identity while preventing precocious terminal differentiation in adult muscle progenitors [21]. In this context, post translational modifications are thought to play an important role controlling Pax7 expression and function in a context dependent manner. Indeed, we have recently described a novel mechanism by which the E3 ubiquitin ligase Nedd4 and the UPS, down regulate Pax7 levels in cells initiating muscle differentiation. Nonetheless, it is not clear how Pax7 protein is regulated in quiescent and proliferating muscle progenitors. Based on previous work, we hypothesized that additional post translational modifications could prevent precocious UPS-dependent Pax7 decline.

Here we demonstrate that Pax7 stability is also regulated by CK2-dependent phosphorylation. Using mass spectrometry analysis, we identified two phosphorylated residues, S201 and S205, in Pax7 protein. Despite the fact that both residues are part of neighbor consensus CK2 motifs, only S201 was directly phosphorylated by CK2 in vitro (Fig 1). Interestingly, proteomic analysis identified phospho-S201, phospho-S205 and phospho-S201-S205 peptides, suggesting that Pax7 could be phosphorylated at S205 in vivo. Whether this phosphorylation represents an intermediate state or has a different effect on Pax7 function remains an open question. In this regard, it has been shown that the Pax7 homolog Pax3 is sequentially phosphorylated at S201, S205 and S209 [30,31]. Interestingly, Pax3 is phosphorylated by CK2 at S205 in proliferating myoblasts [31,32] and this phosphorylation is lost upon differentiation. The same group showed that CK2 phosphorylates Pax3 at S209 only in differentiating cells [30]. How these phosphorylation events regulate Pax3 function and /or stability, remains less clear. Pax3 appears to be phosphorylated at S201 by GSK3β kinase [33], which highlights two key points: i) post translational regulation of Pax3/7 is highly complex and poorly understood, and ii) despite their sequence similarity and partially overlapping expression in muscle cells, Pax7 and Pax3 are regulated independently, potentially by different signaling pathways.

Our results suggest that Pax7 is phosphorylated by CK2 at S201 in proliferating myogenic progenitors. Using site directed mutagenesis, we studied the effect of abolishing (or mimicking) Pax7 phosphorylation. Based on our previous work, we specifically studied the potential effects at the levels of i) sub cellular localization, ii) ability to repress MyoD, iii) transcriptional activity and iv) protein expression. Nuclear expression pattern and transcriptional activity did not differ significantly between Pax7 and Pax7 phospho-mutants. Consistently, all Pax7 mutant proteins were capable of repressing MyoD dependent myogenic conversion of the mesenchymal cell line C3H10T1/2 (Fig 2B). Myogenin induction, cell fusion (myotube formation), MyHC expression, etc., are consequence of the ectopic MyoD transcriptional activity in C3H10T1/2 cells. While this strategy does not pretend to dissect at which particular step the Pax7 mutants are opposing MyoD, we observed decreased myotube formation concomitantly with expression of myogenic markers such as MyHC (Fig 2B) suggesting inhibition of the myogenic progression after myogenic commitment [19,20]. Interestingly, disruption of S201 (Pax7-AS) or S201/205 (Pax7-AA) consistently resulted in lower expression levels when compared to the Pax7-WT protein (Fig 2C). These results suggested that CK2 could regulated Pax7 protein stability. Accordingly, acute CK2 inhibition induced a significant decrease in Pax7 and Pax7 phospho-mutant levels (Fig 3A). Similar results were obtained when analyzing the effect of CK2 inhibition on endogenous Pax7 levels in C2C12 myoblasts (Fig 3B and 3C). Analysis of Pax7 mRNA levels in C2C12 (qPCR) showed no significant changes upon CK2 inhibition, further supporting a role for CK2 at the post translational regulation of Pax7. Noteworthy, RT-PCR analysis of Pax7 mRNA levels in adult primary myoblasts treated with the CK2 inhibitor TBB, showed similar results (Fig 3D) which highlights the physiological relevance of these results. It was not possible to perform a full qPCR analysis in this particular setting due to technical limitations impacting our cell number yields after myoblast isolation and treatments. However, as discussed bellow, functional effects derived from CK2 inhibition over Pax7 expression and myoblasts fate were also replicated in these conditions, further supporting our results.

Since we recently showed that Pax7 was down regulated by the UPS in differentiating myogenic cells, we hypothesized that CK2 inhibition resulted in ectopic Pax7 ubiquitination and proteasomal degradation. Reduction in Pax7 levels upon CK2 inhibition was prevented by concomitant proteasome inhibition (Fig 4A), while inhibition of CK2 activity resulted in a ~2-fold increase of Pax7 ubiquitination (Fig 4B). This observation was further supported by BiFC assays, which also indicated that Pax7 ubiquitination was increased upon CK2 inhibition or Pax7-AA phospho-mutant expression (Fig 4C). Most importantly, CK2 inhibition in proliferating primary adult myoblasts, induced precocious myogenin induction (Fig 5) and therefore accelerated commitment to terminal differentiation. These observations are consistent with our previous findings [21] and suggest that CK2-mediated Pax7 phosphorylation mediates access to the UPS machinery, perhaps in a time and/or extrinsic dependent manner. In order to describe the underlying mechanism in more detail, we performed siRNA mediated CK2-knockdown experiments, but observed mixed effects which cannot be clearly separated: unlike the acute pharmacological inhibition, cultured myoblasts were maintained for ~48–72 h in other to observe significant CK2 down regulation. As described by many groups, CK2 has a plethora of targets affecting many cellular processes, one of them being cell cycle progression. We observed a severe proliferation effect in CK2-siRNA treated myoblasts, which perturbed the levels of not only Pax7 but of other relevant proteins (data not shown), therefore we reasoned that acute pharmacological inhibition would be a more controlled (and less disruptive) strategy to pursue. Although we observed similar results utilizing two non-related CK2 inhibitors, we cannot categorically discard the non specific inhibition of additional kinases. Both TBB and TBCA have been tested extensively in HeLa and Jurkat T-cell lines [34,35], effectively inhibiting CK2 activity at lower concentrations (10 and 5 μM, respectively) than those used in the current study (100 and 50 μM, respectively). However, given the wide range of concentrations reported in the literature, it is likely that the effective concentration for CK2 inhibition is highly dependent on the cell context. For example, Canton DA, et al. (2005) and Duncan JS, et al. (2008), utilized TBB in C2C12 cell cultures for >18 h, even at 75 μM [36,37]. Importantly, Dick SA., et al. (2015) treated adult primary myoblasts with 50 μM TBB for up to 72 h and showed specific caspase 3-dependent down regulation of Pax7 levels [29] (discussed bellow). Given this particular scenario, we consider the TBB concentrations (75–100 μM) and incubation times (6–12 h) used in this study, to be with in the reported range for muscle cells. An inducible shRNA expression system to allow controlled CK2 down regulation, may prove useful to clarify this point.

Key aspects of our model find strong support in the recently published work from Dick and cols. [29]. That study showed that Pax7 is cleaved by caspase-3, rendering Pax7 inactive. This observation has profound implications since inhibition of caspase-3 activity disrupts muscle differentiation, with a concomitant increase in the self-renewing SC population. Conversely, caspase-3 activation leads to a decline of active Pax7 and commitment to differentiation. Our own work previously suggested that Pax7 was regulated by the UPS and caspase-3 dependent cleavage [38], most likely acting in parallel to reinforce the non-reversible commitment to terminal differentiation. Moreover, Dick and cols. showed that CK2-directed phosphorylation of Pax7 at S201, attenuates caspase-directed cleavage, highlighting the physiological relevance of Pax7 phosphorylation in the regulation of SC fate.

Several results presented here suggest that CK2-directed phosphorylation stabilizes Pax7 primarily by decreasing its ubiquitination and proteasome-dependent degradation. Additionally, we evaluated the status of caspase-3 activity upon CK2 inhibition in in primary myoblasts maintained in proliferation culture medium, by immunofluorescence. No significant activated caspase-3 signal was detected under normal proliferating culture conditions (<3% (+)/total cells), whereas activation was evident upon apoptosis induction by increasing concentrations of H_2_O_2_ (30–40%; data not shown). We are currently investigating the specific molecular events affected by Pax7 phosphorylation and how they affect UPS-dependent regulation, which may provide a mechanism to conciliate the molecular consequences of CK2-mediated Pax7 phosphorylation presented in both studies. Such studies will benefit from the use of genetic models, such as cell-specific/inducible CK2 knockouts mice, given the low abundance of satellite cells in the muscle tissue and the necessity of evaluating the specific role of CK2 in an inhibitor-independent manner. Additionally, uncovering the signaling controlling CK2 activity will be key to better understand the differential regulation of Pax7 in quiescent, activated and differentiating muscle progenitors. Most importantly, however, is that collectively the results presented here and work by others underscore the critical role of Pax7 post translational regulation for SC fate, and thus, muscle regeneration and long-term maintenance.
